# Pre-sleep protein supplementation after an acute bout of evening resistance exercise does not improve next day performance or recovery in resistance trained men

**DOI:** 10.1080/15502783.2022.2036451

**Published:** 2022-04-04

**Authors:** Michael J. Ormsbee, Patrick G. Saracino, Margaret C. Morrissey, Jaymie Donaldson, Liliana I. Rentería, Andrew J. McKune

**Affiliations:** a Institute of Sports Science and Medicine, Department of Nutrition and Integrative Physiology, Florida State University, Tallahassee, USA; b School of Health Sciences, Discipline of Biokinetics, Exercise and Leisure Sciences, University of KwaZulu-Natal, Durban, South Africa; c Korey Stringer Institute, University of Connecticut, Storrs, USA; d Research Institute for Sport and Exercise Science, University of Canberra, Canberra, ACT, Australia

**Keywords:** Protein, timing, pre-sleep, athletes, recovery, resistance training, strength

## Abstract

**Background:**

To evaluate the effect of pre-sleep protein supplementation after an acute bout of evening resistance training on next day performance and recovery the following day in physically active men.

**Methods:**

Eighteen resistance trained men performed a single bout of resistance exercise then received either a pre-sleep protein (PRO) supplement containing 40 g of casein protein (PRO; *n* = 10; mean ± SD; age = 24 ± 4 yrs; height = 1.81 ± 0.08 m; weight = 84.9 ± 9.5 kg) or a non-caloric, flavor matched placebo (PLA; *n* = 8; age = 28 ± 10 yrs; height = 1.81 ± 0.07 m; weight = 86.7 ± 11.0 kg) 30 min before sleep (1 h after a standard recovery drink). Blood samples were obtained pre-exercise and the following morning (+12-h) to measure creatine kinase and C-reactive protein. Visual analog scales were utilized to assess perceived pain, hunger, and recovery. One-repetition maximum (1RM) tests for barbell bench press and squat were performed pre-exercise and the following morning (+12-h). Statistical analysis was performed using SPSS (V.23) and *p *≤ 0.05 was considered statistically significant.

**Results:**

There were no significant differences between the groups in next morning performance or muscle damage biomarkers. However, pre-sleep PRO resulted in a lower perception of hunger that approached significance the following morning when compared to PLA (PRO:43.6 ± 31.2, PLA: 69.4 ± 2.22; 95% C.I. = −53.6, 2.0; *p* = 0.07; *d* = 0.95).

**Conclusions:**

Following an evening bout of exercise, pre-sleep PRO did not further improve next morning muscle damage biomarkers or maximal strength performance in resistance trained men compared to a non-caloric PLA. However, there may be implications for lower perceived hunger the next morning with pre-sleep PRO consumption compared to PLA.

## 1. Introduction

Post-exercise protein has been widely accepted as a nutritional strategy to increase muscular protein synthesis (MPS) and improve post-exercise recovery [1]. Several factors have been suggested to mediate the response of protein ingestion including dose [2,3], type [4–6], distribution [7], and timing [8,9].

Regarding timing, pre-sleep protein ingestion provides an additional opportunity to consume protein. Early work in the area of ‘pre-sleep feeding’ indicated that pre-sleep protein is normally digested and absorbed in both younger [10] and older populations [11]. When 40 g of labeled casein was ingested by young men, MPS was elevated compared to non-caloric placebo [10]. Pre-sleep protein alone has been shown to increase MPS. However, this response is amplified when an acute bout of resistance exercise (RE) is performed in the evening (i.e. in close proximity to pre-sleep protein ingestion) [8,12]. In fact, myofibrillar protein synthesis rates were 37% greater with pre-sleep casein ingestion in combination with nighttime resistance training compared to pre-sleep casein ingestion alone [8]. Snijders et al. was the first to show an increase in muscle size and strength following 12 weeks of evening resistance training when a casein protein (27.5 g) and carbohydrate (CHO; 15 g) beverage was consumed pre-sleep [12].

Pre-sleep protein has also been reported to improve muscle recovery following an evening bout of damaging exercise [13,14]. West et al. provided 25 g whey or calorie matched CHO control to young men immediately following a nighttime bout of whole-body resistance training (2000 h) [14]. These authors reported improvements in net protein balance, maximal isometric voluntary contraction, repetitions to failure, and Wingate power output in the 24-hour recovery period with pre-sleep whey protein consumption compared to CHO control [14]. Similarly, in the 60-h following nighttime professional soccer matches, Abbott et al. reported improvements in maximal jump height, reactive strength index, and muscle soreness when 40 g of pre-sleep casein protein was ingested compared to an iso-energetic carbohydrate supplement [13].

Interestingly, a pattern is emerging in the literature where the timing of exercise is an important factor in the efficacy of pre-sleep protein consumption. The benefit of pre-sleep protein has been shown with evening exercise [10,12–14]; however, pre-sleep protein has not shown benefit when the bout of exercise is performed in the morning or early afternoon (i.e. hours away from the exercise stimulus) [15–18]. Following 12 weeks of resistance training in the morning (between 0800 and 1100 h) in older men, pre-sleep protein resulted in similar improvements in quadriceps cross-sectional area, muscle strength, and physical performance compared to placebo [16]. Apweiler et al. subjected young men and women to a morning (between 0730 and 0900 h) bout of 100 drop jumps and reported no differences between pre-sleep casein protein or carbohydrate treatments for maximal isometric voluntary contraction, jump height, or muscle soreness [18]. Additionally, no benefit was reported in trained runners when consuming 0.5 g/kg whey protein compared to calorie matched carbohydrate pre-sleep for 5-km time trial performance, creatine kinase (CK), lactate dehydrogenase, or myoglobin during a 1-week endurance training camp regimen with morning and afternoon training sessions [17]. Thus, it appears that exercise timing plays a critical role in the efficacy of pre-sleep protein consumption, with pre-sleep protein having benefit when consumption is in close proximity to an exercise stimulus.

We have previously shown that pre-sleep chocolate milk consumption altered the next day metabolism but not exercise performance in trained female runners [19]. Additionally, pre-sleep whey and casein protein likely altered the next morning metabolism with no significant effects on the next morning resistance training volume in resistance trained women [20]. Whey protein has been reported to have small to moderate beneficial effects compared to CHO at 24 h following nighttime RE for maximal voluntary contraction and repetitions to failure in trained, young men [14]. Similarly, casein protein had likely beneficial effects on countermovement jump and most likely enhanced reactive strength index performance in professional soccer athletes 12-h following nighttime soccer matches compared to CHO. However, it is unknown if pre-sleep protein ingestion improves next day maximal strength performance or indirect markers of muscle damage (CK) and inflammation (C-reactive protein (CRP)), using traditional movements like the squat and bench press in active, resistance-trained men. While it has been shown that resistance exercise induces a rise in circulating CK, albeit demonstrating high interindividual variability [21], the effect of acute bouts of resistance training on CRP are equivocal and there is potential for rise in CRP when there is muscle damaging exercise [22]. Therefore, the purpose of the present study was to investigate the effects of nighttime protein supplementation compared to a non-caloric placebo following a bout of evening resistance-type exercise on next day strength performance and recovery in physically active men. We hypothesized that pre-sleep protein supplementation would attenuate decrements in one repetition maximum (1RM) strength performance and improve markers of muscle damage and inflammation compared to placebo 12-h post-RE.

## 2. Methods

Twenty resistance-trained men were recruited from various athletic training centers across the province of KwaZulu- Natal, South Africa. Participants between the ages of 18 and 45 years old who performed a minimum of 5 h of resistance training per week for the past year were included in the study. Participants were excluded if they had: (1) a history of lower or upper extremity injuries that would be aggravated by the requirements of the study or inhibit performance; (2) an allergy to milk or dairy; (3) regular use of anti-inflammatory drugs or medication; (4) previous and current use of any performance enhancing drugs; and (5) the use of massage, cryotherapy, compression garments, and any other recovery enhancing activities for the duration of the study. Consumption of caffeine and alcohol was prohibited throughout the trial as well as any other supplementation with protein or other ergogenic aid for 2 days prior to and for the duration of the trial.

Informed written consent was obtained from each participant. They were briefed on all experimental procedures and risks associated with the project and a full medical history questionnaire was completed before the commencement of the study. Ethical clearance for the study was obtained the University of KwaZulu Natal’s Biomedical Research Ethics Committee (REF: BFC 354/13).

### 2.1. Study design

The study employed a randomized, placebo-controlled, double-blind design, where participants were randomly assigned to ingest either 40 g of casein protein (PRO; Dymatize) or a non-caloric, flavor-matched placebo (PLA; Pepsi Co.) mixed with 450 mL of water before sleep. A 40 g bolus was chosen as this has been reported as the minimum required dose to improve overnight myofibrillar protein synthesis [8]. Participants were randomly assigned to each group using an online randomization generator (www.randomization.com). Participants visited the laboratory 1-week prior to exercise testing for baseline testing and a familiarization trial (visit 1). Participants then came to the laboratory on visit 2 for the experimental exercise session and visit 3 for the next morning testing (12-h following the exercise session).

### 2.2. Baseline testing and familiarization (Visit 1)

Visit 1 occurred in the morning (730–930 h), one week prior to visit 2, and a minimum of 1-h post each participant’s regular breakfast. Body weight was measured using a digital scale, height was measured using a wall-mounted stadiometer, and Harpenden skinfold calipers were used to assess the body composition. Seven skinfold sites (chest, triceps, mid-axilla, subscapular, supra-iliac, abdominal, and thigh) were measured to calculate body density and body fat percentage [23]. Each site was measured twice, and a third measurement was performed if measurements were not within 2 mm. With the inclusion criteria specifying the necessity of experience in resistance training, the participants were familiar with the required movements for the 1RM tests (barbell squat and bench press). Based on verbal confirmation, they regularly performed these movements in training and had performed 1RM testing previously using these movements as part of performance testing for program adjustment. However, participants were familiarized with the exercise protocol and were provided instructions on safe lifting technique for the lifts to ensure appropriate lifting technique and limit learning effects. Baseline 1RM testing for both squat and bench press were subsequently conducted. The participant’s 1RM was defined as the highest weight moved one time through the full range of motion using proper form.

Participants were instructed how to record dietary intake by a sports dietitian for the three days leading up to visit 2 (including their intake on the day of visit 2). Participants were required to abstain from caffeine, alcohol, exhaustive physical labor and exercise for 24 h prior to the 1RM testing session and for 3 days prior to visit 2. Compliance for the physical activity was assessed using an activity questionnaire.

### 2.3. Experimental testing (visits 2 and 3)

**Figure 1.**
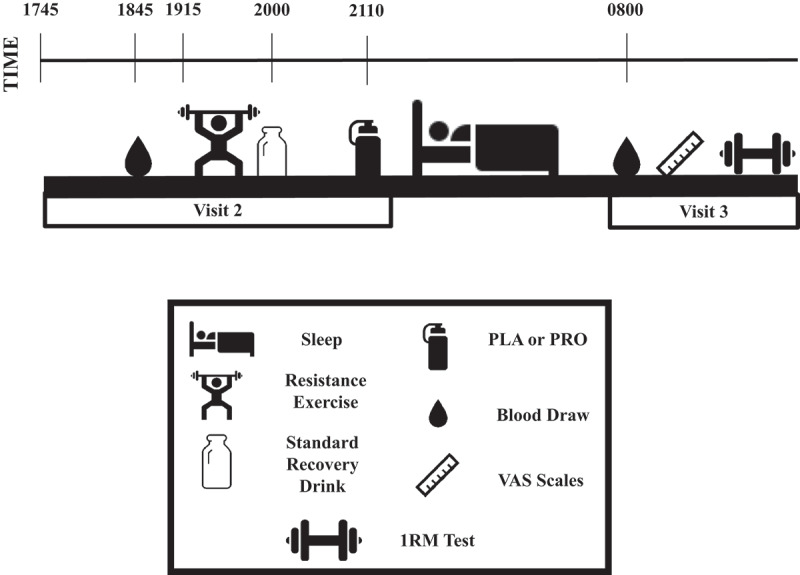
Visit 2 and 3 timeline.

Experimental testing days are presented in Figure 1. Participants reported to the laboratory in the evening (1745 h) on visit 2 after an early dinner (1700 h) that was recorded in the three-day dietary record. Participants were asked to remain in the supine position for ~1-h prior to the pre-RE blood sample. Capillary and venous blood samples were drawn to assess CK and high-sensitivity C-reactive protein (hsCRP), then the exercise protocol was performed at 1915 h as described below. All participants consumed a recovery drink with 60 g CHO from maltodextrin (Pepsi Co.) and 20 g whey hydrolyzate PRO (Dymatize) immediately following the exercise bout. One hour following consumption of the post-exercise recovery drink (2110 h), participants consumed their assigned pre-sleep drink under researcher supervision prior to returning home to ensure compliance of supplement consumption.

PLA: placebo, PRO: pre-sleep protein (40 g), VAS: visual analog scale, 1RM: 1 repetition maximum.

All participants were instructed to have two bananas in the morning before returning to the laboratory for laboratory visit 3. Compliance was verbally verified by researchers. Participants were also asked to refrain from caffeine and alcohol and return to the laboratory at 0800 h for a blood sample collection (+12-h). After blood collection, participants rated their perceived hunger, muscular pain in both the legs and upper body, and rate of recovery using visual analog scales (VAS). The VAS were 100 mm in length with ‘not at all’ and ‘extremely’ indicated on the left and right ends of the line, respectively. Participants drew a vertical mark through the line at the position, which they felt corresponded with the way they felt. The distance from the left end of the line to the mark was used for analysis. Lastly, 1RM testing for both squat and bench press were performed.

### 2.4. Exercise protocol

The RE session consisted of eight sets of eight repetitions of weighted barbell back squats and weighted barbell chest press. In both exercises, one set was performed at 55% individual’s 1RM, prior to one at 65% 1RM and then six sets were performed at 75% 1RM. There was a resting period of 2 min between sets and a 5 min rest between exercises. The entire protocol took 45 min to complete. All participants were verbally encouraged throughout the exercise bout to complete the entire program, and water was available ad libitum. This exercise protocol was adapted from an investigation of the effects of protein ingestion before sleep on post-exercise overnight recovery [10].

### 2.5. 1RM testing

The 1RM testing protocol used in this study [24] required participants to be familiar with bench press and squat movements with a weighted barbell. Participants started with 10 repetitions of the movement (squat or bench press) with very light, self-selected resistance as a warm-up set. After a 1 min rest period, weight was increased by 5–10 kg for the upper body, and 15–20 kg for the lower body and 3–5 repetitions were performed for the first set. Participants rested for 2 min before increasing weight again (5–10 kg for the upper body and 15–20 kg for lower body) and completed 2–3 repetitions. A 2–4 min rest was provided prior to the first 1RM attempt, which was approximately 50% of the participant’s estimated 1RM weight. Participants rested for 2–4 min between 1RM attempts. If an attempt was failed, researchers decreased the weight by 2.5–5 kg for the upper body and 7.5–10 kg for the lower body, and another attempt was executed.

### 2.6. Blood collection and processing

Blood samples were collected at pre-RE, and the following morning at 12-h post-RE (+12). Blood for CK was collected via a finger stick using a microcapillary tube under sterile conditions. CK (CK-MM isoform) blood samples (approximately 32 μl) were immediately analyzed using a Reflotron (Boehringer Mannheim GmbH, Germany) blood analyzer, which used a colorimetric assay procedure. If the values were >1500 IU/l, the sample was diluted with equal parts distilled water and re-analyzed immediately. Samples of hsCRP were collected from the antecubital vein in serum vacutainers. After the collection, samples were allowed to clot at room temperature and then centrifuged at 3500RPM at 4°C for 15 min (Sorvall ST16R Multispeed Centrifuge; Thermo Electron Corporation, Needham Heights, Massachusetts). Separated serum was divided into 0.5 mL aliquots and stored at −80°C until analyzed. Concentrations of serum hsCRP were measured using the N Latex hsCRP kit (Behring Diagnostics, Frankfurt, Germany) according to the manufacturer’s instructions. Specimens were mixed with polystyrene particles coated with monoclonal antibodies, and the intensity of light scattering was measured in a Behring nephelometer.

### 2.7. Statistical analysis

An independent *t*-test was used to determine if mean differences existed at baseline for participant characteristics, nutritional intake, and 1RM scores. Cohen’s *d* effect sizes (ES) and 95% confidence intervals (CI) were also calculated for all the outcome measures that were assessed via an independent *t*-test. A two-way analysis of variance (ANOVA) with repeated measures was conducted to determine if CK and CRP were significantly altered by pre-sleep PRO. All measurements had two groups (PRO, PLA) and two time points (pre-RE, +12-h). Significant main effects were further investigated using Bonferroni pairwise comparisons. If the sphericity is violated, Greenhouse–Geisser corrections are utilized. All group × time interactions and main effects are reported as (*F*_df treatment, df error_) = *F* statistic, *p*-value, partial eta squared (*η*_*p*_^2^). Significant main effects were further investigated using Bonferroni pairwise comparisons. For *η*_*p*_^2^, effect size values were qualified as follows: small = 0–0.02, medium = 0.02–0.13, large = 0.13–0.26. The level of significance was set at *p* ≤ 0.05 for all statistical tests. Statistical analysis was performed using the IBM SPSS Statistics for Windows, Version 25.0 (Armonk, NY: IBM Corp).

## 3. Results

### 3.1. Participant characteristics

Twenty participants were screened and performed baseline testing. The two participants dropped out of the study due to injury and illness, leaving 18 participants for final analysis with an equal percentage of rugby players and Crossfitters in each group (PRO *n *= 10, 5/10 rugby; PLA *n* = 8, 4/8 rugby). There were no significant differences at baseline between groups for age, height, weight, body mass index (BMI), body fat %, absolute or relative bench press strength, or relative squat strength. However, there was a significant difference in absolute squat strength with PLA having a greater 1RM squat at baseline compared to PRO (*p* = 0.044; Table 1).

**Table 1.** Participant characteristics

	PRO (*n* = 10)	PLA (*n* = 8)	*p*-value
Age, yr	24 (4)	28 (10)	0.27
Height, cm	181 (8)	181 (8)	0.89
Weight, kg	84.9 (9.5)	86.7 (10.9)	0.72
BMI kg/m^2^	25.8 (1.6)	26.5 (2.0)	0.43
Body Fat, %	7.5 (2.2)	10.0 (3.1)	0.07
Squat 1RM, kg	140.4 (20.7)	162.4 (21.8)	0.04*
Squat 1RM, kg/bm	1.67 (0.26)	1.89 (0.29)	0.11
Bench Press 1RM, kg	109.8 (25.0)	115.3 (18.7)	0.61
Bench Press 1RM, kg/bm	1.28 (0.21)	1.35 (0.27)	0.57

Data are mean (SD), bm: body mass in kg, BMI: body mass index, 1RM: 1 repetition maximum, relative strength: 1RM (kg)/body weight (kg), * indicates *p* ≤ 0.05.

### 3.2. Nutritional intake

Nutritional intake for the 3 days prior to experimental testing are reported in Table 2. There were no significant differences between groups for calories, CHO (g), fat (g), PRO (g), or PRO (g/kg).

**Table 2.** Nutritional intake

	PRO (*n* = 10)	PLA (*n* = 8)	Total	*p-*value
Calories, kCal	3057 (777)	2729 (1155)	2903 (956)	0.49
CHO, g	225 (90)	210 (132)	218 (108)	0.78
Fat, g	78 (22)	70 (26)	74 (24)	0.49
PRO, g	162 (27)	138 (35)	151 (32)	0.13
PRO, g/kg	1.9 (0.3)	1.6 (0.4)	1.7 (0.3)	0.11

These data also include all supplements. Data are represented as mean (SD).

### 3.3. 1RM squat and bench press

**Figure 2.**
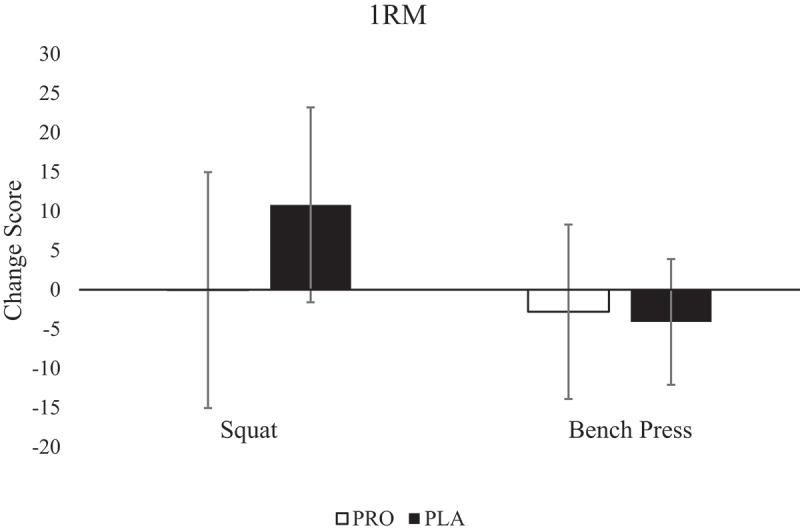
1RM.

Since the baseline strength was significantly different, data were converted to a change score (+12-h 1RM – Pre-RE 1RM), and independent samples *t*-tests were run. There were no significant differences between groups for change score in squat 1RM (PRO: −0.4 ± 15.0 kg; PLA: 10.8 ± 12.4 kg; *p* = 0.112; CI: −25.2, 2.9) or bench press 1RM (PRO: −2.8 ± 11.1 kg; PLA: −4.1 ± 8.0 kg; *p* = 0.783; CI: −8.6, 11.2) (Figure 2). Interestingly, only four participants for squat and nine participants for bench press had a reduced 1RM at +12-h compared to the baseline.

One repetition maximum (1RM) represented in both (A) squat and (B) bench press. Data are presented as mean ± SD. Pre-RE, pre-resistance exercise; +12-h, 12 hours post-resistance exercise. * indicates statistical difference between groups in that specific lift at Pre-RE and +12-h.

### 3.4. Visual analogue scales

Data for pain and hunger VAS are presented in Table 3. There was a trend for lower next day hunger for PRO compared to PLA (22.7 ± 20.7 mm, 28.4 ± 20.8 mm; *p *= 0.067; CI: −5.36, 2.0). There were no differences between groups for perceived recovery, pain in legs, or pain in the upper body.

**Table 3.** Pain, hunger, and recovery

	PRO	PLA	*p*-value	CI	Cohen’s *d*
Pain – Legs	22.7 (20.7)	28.4 (20.8)	0.57	−2.66, 1.52	0.27
Pain – UB	15.8 (15.2)	22.3 (19.3)	0.44	−2.36, 1.07	0.37
Hunger	43.6 (31.2)	69.4 (22.2)	0.07	−5.36, 0.20	0.95
Recovery	69.7 (23.9)	59.6 (21.3)	0.37	−1.28, 3.29	0.45

UB, upper body. Mean (SD). Cohen’s *d* = (*M*_2_–*M*_1_)/*SD*_pooled_; *SD_pooled_* = √((SD_1_^2^ + SD_2_^2^)/2).

### 3.5. Recovery and inflammation biomarkers

There were no significant differences at baseline between the groups for CK (*p* = 0.73) or CRP (*p* = 0.46). There were no significant time × treatment interactions for CK (*F*_1, 16_ = 0.353, *p* = 0.56, *η*_*p*_^2^ = 0.022) or the main effect of group (*F*_1,16_ = 0.016, *p* = 0.90, *η*_*p*_^2^ = 0.001). However, there was a significant time effect (*F*_1,16_ = 12.642, *p *= 0.003, *η*_*p*_^2^ = 0.441) with CK concentrations significantly greater at +12-h compared to pre-RE (*p* = 0.003). For CRP, four participants had concentrations below the assay sensitivity, thus were not included in the analysis (*n* = 14). There were no significant time × treatment interactions for CRP (*F*_1, 12_ = 0.198, *p* = 0.66, *η*_*p*_^2^ = 0.016), main effects of group (*F*_1,12_ = 0.617, *p* = 0.447, *η*_*p*_^2^ = 0.049), or time (*F*_1,12_ = 0.044, *p* = 0.84, *η*_*p*_^2^ = 0.004).

## 4. Discussion

The novel findings of this investigation were that pre-sleep protein supplementation following a bout of evening RE did not improve next day RE performance, muscle recovery, or perceived hunger in the 12-h recovery window.

There were no significant differences between PRO and PLA for squat or bench press 1RM 12-h following RE in the present study. This disagrees with previous investigations reporting the benefit of pre-sleep protein on next day exercise performance [13,14]. It is possible that differences in performance measures explains the differences in findings. West et al. utilized repetitions to failure, counter movement jump, and maximal voluntary contraction and Abbott et al. utilized counter movement jump and reactive strength index to assess performance [13,14]. Another likely explanation is the lack of performance reduction resulting from the exercise bout in the present study and different methods in attempts to induce muscle damage. Previous reports have suggested that maximal voluntary contraction is the best way to indirectly quantify muscle damage, however 1RM has greater external validity, which is why it was selected [24]. In the present study, only four participants for squat and nine participants for bench press had reduced performance the following day. While the RE protocol utilized in the present study is based on the previous literature [14], the results may be indicative that it was not sufficient to induce muscle damage in most participants. This may be due to differences in training modalities within our participants as some were professional rugby players, while others were Crossfitters, leading to varying rates of muscular recovery. Further, the previous studies from which the present protocol was derived assessed different populations. In the present study, participants engaged in activities that were similar to the muscle damage protocol, possibly making them more adapted to the bouts of exercise and less likely to experience next day performance detriments. However, because it was a protocol that had exercises familiar to the participants, it has a higher degree of external validity. Additionally, because professional athletes were included in the present study, a more conservative approach was taken to decrease risk of injury or interrupt their normal training regimen. Studies investigating pre-sleep PRO supplementation on muscle recovery and performance have shown equivocal results. Interestingly, a trend has emerged suggesting the benefit of pre-sleep protein with nighttime exercise, while no benefit is reported with morning exercise. Conversely, while the present study performed RE at night, there was no benefit to recovery. This is likely due to both groups receiving the same standard recovery drink immediately post-exercise, which may have met their recovery needs prior to receiving their pre-sleep nutrition drink. It is also possible that sufficient muscle damage was not induced by the exercise protocol, due to the fitness level of the participants.

The current study, as well as others, has primarily examined muscle damage and inflammatory biomarkers (CK and hsCRP) following protein consumption and RE. After strenuous, novel exercise, serum CK has been shown to dramatically rise and peak at 24–120 h post exercise and gradually return to baseline over time [25,26]. Protein supplementation has been previously reported to attenuate CK post-exercise [27,28]. Brown et al. provided two 20 g doses of whey protein hydrolyzate or carbohydrate for 4 days following a strenuous exercise protocol for active females. Serum CK concentrations were elevated post-exercise and protein attenuated increases at 48-h compared to carbohydrate [28]. Xia et al. provided 25 g of protein split into morning and evening boluses for 14 days prior to exercise and for 4 days following the exercise to untrained college males. It was reported that CK concentrations were reduced at 24, 48, and 72 h in the protein group compared to carbohydrate [27]. It should be noted that this study utilized an oat protein. Future work should examine the effects of protein sources on muscle damage indices following strenuous exercise. While the present study does not agree with the findings of Brown et al. and Xia et al., as we noted no attenuation in the rise of post-exercise CK concentrations between PRO and PLA groups [27,28], others have reported no benefit of protein supplementation on CK concentrations [29–31]. Additionally, in the current study, there appeared to be high variability of CK concentrations between all individuals at each time point, which may have influenced changes over time. CK levels have been shown to have immense inter-subject variability when exposed to the same exercise stimulus [25,32–34]. However, CK was increased following RE with no differences between groups. Although CK is often utilized as an indirect marker of muscle damage as it is an intramuscular protein, the present study may not have observed changes due to it also reflecting physiological and metabolic load of the RE without specific damage. While CK may be indicative of complex interactions associated with the energy status and scale of muscle disturbance, rates of CK clearance also impact serum levels [35]. Additionally, the energy sensing enzyme AMP-activated protein kinase (AMPK) has been shown to regulate the CK levels in cytosol [26]. This process facilitates the phosphorylation of CK, which allows for CK to be released from the cytosol. Because the participants in the present study were well trained, it is possible that CK was elevated post-exercise due to the metabolic load of the exercise session rather than due to the muscular damage induced from the bout of exercise, as indicated by the lack of soreness and sustained next-day performance. While it is possible that measuring CK at later timepoints (>12 h post) may have resulted in observable differences, it is important to note that the research logistics were secondary to the professional athletes’ normal training regimen and playing schedule.

Similarly, there were no group or time effects in hsCRP concentration. While some studies have reported increased CRP concentrations post-exercise [27,36], others have reported no change [33,37] which agrees with the present study. For example, Cipyran et al. (2015) examined the recovery pattern of CRP and interleukin-6 (IL-6) after maximal exercise (continuous or intermittent all-out exercise) and reported no changes in CRP despite changes in CK, swelling, and soreness [37]. It has been suggested that the magnitude of the exercise stimulus is a key factor in the response or adaptation to exercise, as CRP tends to only increase following a strenuous or prolonged bout of exercise or exercise resulting in muscular injury [38]. Thus, it is possible that the exercise stimulus in the present study was insufficient to induce increases in CRP concentrations. Additionally, previous research suggests that CRP only increases post-exercise in high CK responders – so not only does the exercise stimulus need to be sufficiently strenuous, but it also needs to be in high CK responders to observe differences in CRP [39]. Further, it has been suggested that habitual exercise or training can lead to lower basal levels of circulating inflammatory markers, as well as a reduction in the inflammatory response to acute exercise [40]. Since our population was well-trained men, it is possible that no increases were noted due to the training status of our population.

Some investigations have examined subjective assessments of satiety after protein consumption after a prolonged post-prandial period such as sleep [41–45]. Lehy et al. (2018), Kinsey et al. (2014), and Ormsbee et al. (2014) reported an increased satiety in obese individuals after pre-sleep protein intake; however, in athletes, even less is known [42,43]. While not statistically significant, the present study noticed a trend for increased next day satiety. However, this relationship is not consistently observed in the literature. Madzima et al. reported no differences between casein, whey, or carbohydrate consumed pre-sleep on appetite measures in young, physically active men [45], which contrasts findings from the present study where subjective measures of appetite were improved in athletic males. It should also be noted that none of these investigations examined whether improvements in appetite resulted in changes in energy balance. Lay et al. reported a similar energy intake at an ad libitum breakfast and during the 24-h following a fluid skimmed milk bedtime snack containing either 10 g or 30 g of protein in overweight, untrained men compared to placebo [46]. Thus, pre-sleep protein may improve perception of hunger and appetite, but may not reduce next day caloric intake.

## 5. Limitations

A major limitation of the present study is the lack of standardized diet. While efforts were made to record dietary intake, food logs have inherent reliability concerns. Future work investigating pre-sleep protein supplementation should standardize nutrition. Secondly, while the RE bout utilized in the present study was practical, it was likely not intense enough to cause sufficient muscle damage in our participants. Future work should utilize more strenuous exercise protocols to examine muscle damage. Lastly, the present study did not utilize the strength requirement for inclusion into the study, and thus did not account for differences in physical performance at baseline. Future work should utilize an assessment of baseline strength for inclusion into the study or stratify by strength.

## 6. Conclusion

Pre-sleep PRO did not further improve the next morning biomarkers of recovery or maximal strength performance in recreationally trained young men that consume adequate daily protein for athletes compared to a non-caloric placebo. Further research is warranted to examine whether pre-sleep protein supplementation can augment next day recovery and performance and the influence that time of day of exercise plays in this paradigm.

## Data Availability

The datasets during and/or analysed during the current study available from the corresponding author on reasonable request.
